# Validation of real-time reverse transcription polymerase chain reaction to detect virus titer and thermostability of Newcastle disease live virus vaccine

**DOI:** 10.14202/vetworld.2018.1597-1603

**Published:** 2018-11-20

**Authors:** Pangkaj Kumar Dhar, Avijit Dutta, Avijit Das, Mohammad Shah Jalal, Himel Barua, Paritosh Kumar Biswas

**Affiliations:** Department of Microbiology and Veterinary Public Health, Chittagong Veterinary and Animal Sciences University, Khulshi, Chittagong-4225, Bangladesh

**Keywords:** LaSota strain, Newcastle disease, real-time reverse transcription polymerase chain reaction, thermostability, vaccine, validation

## Abstract

**Background and Aim:**

Newcastle disease is one of the most common diseases affecting poultry in Bangladesh. The disease can cause up to 100% mortality but is preventable if birds are timely and properly vaccinated with a vaccine of standard virus titer. Different live vaccines are commercially available in the country - most, if not all, are produced using lentogenic strains of the virus with variable virulence. One of the disadvantages of these vaccines is that they are not stable at high environmental temperature, and therefore, a proper cold chain must be maintained during transportation and storage. Information on how long these vaccine viruses can withstand environmental temperature, which is near the vicinity of 37°C in the summer season in Bangladesh, is scanty. The aim of this research was to measure the effect of temperature on virus titer of live ND virus vaccines and to develop a real-time reverse transcription polymerase chain reaction (rRT-PCR) standard curve to indirectly determine hemagglutination (HA) titer of virus by this highly sensitive method.

**Materials and Methods:**

In this study, thermostability of five commercial live vaccines containing LaSota, F, Clone 30, and B1 type LaSota strains was observed for up to 35 days keeping them at 37°C. From the most thermostability yielding sample, two rRT-PCR standard curves were developed: (1) By plotting the cycle threshold (C_T_) values as obtained from 10-fold serial dilutions up to 10^−3^ against their corresponding log (to the base 10) dilutions and (2) by plotting the C_T_ values obtained from serial HA dilutions up to 2^−4^ against their corresponding HA titer dilutions. The PCR efficiencies based on which the graphs were fitted were also evaluated.

**Results:**

The vaccine from the LaSota strain withstood 37°C for 35 days with a gradual declination of HA titer over time, and this vaccine also had the highest initial HA titer, which was 2^11^. The vaccine made from F strain was inactivated quickly, and it had the lowest HA titer at the beginning of the study. The first standard curve developed can be used to assess the level of virus titer in a diluted sample compared with the titer in the original undiluted vaccine preparation by plotting the C_T_ value obtained from the dilution by rRT-PCR. The second standard curve can be used to calculate the HA titer of a vaccine dilution by plotting the C_T_ value as obtained from the dilution by rRT-PCR.

**Conclusion:**

The regression equations for the first and second graphs were y=−3.535x+14.365 and y=−1.081x+13.703, respectively, suggesting that, for every 3.53 cycles, the PCR product would have increased 10 times and 2 times for every 1.08 cycles, respectively, indicating nearly (but not exactly) 100% PCR efficiency.

## Introduction

Newcastle disease (ND) is considered as one of the most important infectious diseases of poultry due to its potential for devastating loses. It is one of the leading causes of mortality in chickens due to respiratory symptoms in poultry that occurs worldwide [1,2]. It affects respiratory, nervous, and digestive systems and shows variable symptoms depending on the strain of the virus, species of bird, concurrent disease, and preexisting immunity. ND virus (NDV) is the causal agent of ND and is a member of the genus Avulavirus [3,4] in Paramyxoviridae family. NDV is an enveloped virus which consists of a negative-sense, single-stranded, non-segmented, RNA genome. It is also known as avian paramyxovirus 1 (APMV-1) [5]. Based on pathogenicity in chicken, there are three pathotypes or strains of NDV have been identified, namely highly virulent (velogenic), intermediate (mesogenic), or avirulent (lentogenic) [6]. NDV genome is composed of six genes, and their corresponding six structural proteins are nucleoprotein (NP), phosphoprotein (P), matrix (M), fusion (F), hemagglutinin-neuraminidase (HN), and RNA polymerase (L) [7]. Among them, only the HN and F glycoproteins, important for binding and fusion of the virus to host cells, are known to induce neutralizing antibodies after vaccination that confers protection from morbidity and mortality associated with virulent NDV infection [8-10]. Infection by velogenic strain may cause 80-90% mortality in adult birds [11]. The severity of the disease varies greatly, spanning from peracute case with almost 100% mortality to subclinical with no lesions [12,13].

Vaccination is the most successful tool for the prevention of ND. Improper vaccination may result in the outbreak of ND in chicken [14,15]. All the NDV viruses belong to APMV-1 viruses; therefore, by definition, any NDV vaccine strain should provide protection against morbidity and mortality from any NDV challenge virus [16]. The efficacy of a live vaccine administered is significantly dependable on the virus titer in the vaccine [17,18]. Environmental temperature has a negative impact on persisting live virus titer [19]. Thus, maintaining the cooling chain during transportation and storage of a live ND vaccine is a prerequisite for a satisfactory immune response while administered in poultry [20].

However, very little is known on the degree of thermostability and effect on environmental temperature on virus strains used as live ND vaccines in Bangladesh. The titer of virus in a vaccine can be roughly assessed by hemagglutination (HA) test [17,21] but can more reliably be estimated with other highly sensitive testing, such as real-time reverse transcription polymerase chain reaction (rRT-PCR) [22]. To make a relationship between these two tests, validation is required to develop rRT-PCR standard curve to measure the level of virus titer in terms of HA unit in a live vaccine suspension.

The aim of this research was to measure the effect of temperature on virus titer of live ND virus vaccines and to develop a real-time reverse transcription polymerase chain reaction (rRT-PCR) standard curve to indirectly determine hemagglutination (HA) titer of virus by this highly sensitive method.

## Materials and Methods

### Ethical approval

Experiment was not done on any living animal, so, no ethical approval was required.

### Samples

Five ND live virus vaccines from different lentogenic strains available commercially in Bangladesh were chosen for the present study. The vaccines were collected from local pharmaceutical shops except sample 1, which was from the Department of Livestock Service, Bangladesh. All the vaccines were carried in ice boxes and stored at 4°C in the laboratory until the experimentation begins. Before further testing of the vaccines, all the vaccines were prepared according to the manufacturer guidelines. 500 doses of vaccines were mixed thoroughly with 1.5 ml of distilled water, and 1000 doses of vaccines were mixed with 3 ml of distilled water just before the experimentation.

### Plate HA test

To check the thermostability of the vaccines, all the samples were run for plate HA test, repeatedly, using 1% chicken red blood cells collected from specific pathogen-free flock [23]. The test was operated on 1^st^, 2^nd^, and 3^rd^ days consecutively and then followed on every 7^th^ day until the titer downgraded to nil. After the test on 1^st^ day, vaccines were kept in an incubator at 37°C until the end of the test. Three different batches of the vaccines were tested with the same procedure in three different times.

### Determination of thermostability

Result obtained from every test was recorded in a table and thermostability of the vaccines was determined by calculating the rate of downgrading of the HA titer. The vaccine that showed the longest period of thermostability in the initial experiment was chosen to develop an rRT-PCR standard curve to determine HA titer of a live ND vaccine by performing rRT-PCR.

### Vaccine selection for rRT-PCR

Vaccine sample that showed the longest period of thermostability at 37°C was used for real-time PCR experiment. The PCR was based on a 150 bp, conserved region of the APMV-1 L polymerase gene, in which two probes were included, as per the protocol followed at the UK Animal and Plant Health Agency. Two serial dilutions of the vaccine were made: 10-fold and 2-fold. 10-fold serial dilution was made up to 10^−3^ and 2-fold up to 2^−4^. From each of 10-fold diluted sample, the sample was tested once for detecting the cycle threshold (C_T_) value, while each 2-fold dilution was tested twice to calculate the mean C_T_ value.

### Primers and probes

Two different sets of primer and probes designed for the amplification of RNA polymerase (L) gene were used in rRT-PCR (Table-1) [24].

**Table-1:** PCR primer and probe sequences used to detect L gene of APMV-1 [24].

Primer/Probe	Sequence (5’- 3’)
Forward primer	GAG CTA ATG AAC ATT CTT TC
Reverse primer	AAT AGG CGG ACC ACA TC TG
LproMGB	[6FAM] CCA ATC AAC TTC CC [MGBNFQ]
LproMGB2	[VIC] AAT AGT GTA TGA CAA CAC [MGBNFQ]

PCR=Polymerase chain reaction, APMV-1=Avian paramyxovirus 1

### Preparation of 10-fold and 2-fold dilution

First, 10-fold serial dilution was prepared by adding 100 µl of parent vaccine sample with 900 µl of phosphate-buffered saline (PBS) to make 1000 µl of first 10-fold (10^−1^) dilution. Following the same way, 10^−2^ and 10^−3^ fold dilutions were prepared. In case of 2-fold dilution, 500 µl of vaccine samples were added to 500 µl of PBS, and in the same way, it was repeated to make 2^−1^, 2^−2^, 2^−3^, and 2^−4^ fold dilutions. Counting two from each dilution, a total of eight samples were chosen for final rRT-PCR.

### Extraction of RNA and rRT-PCR

RNA was extracted with the RNase kit (MagMax™-96 Viral RNA Isolation Kit) using the protocol supplied by Ambion Inc. on automatic device Thermo Fisher mL (Thermo Scientific). The extracted RNAs were collected and transferred into sterile Eppendorf tubes as viral RNA extracts and preserved at −20°C until used.

The final amplification process was performed in Thermo Cycler (Applied Biosystems 7500 Fast rPCR system, Foster City, California, USA) in Micro AMP Fast Optical 96-well reaction plate. The components of master mix per 20 µl for this reaction was (for L gene), Molecular grade water (i.e., RNAse-free) 2.75 µl, Qiagen QuantiFast Probe RT PCR buffer mix (w/o ROX) 12.5 µl, 68 µl ROX to 1.7 ml QuantiFast Probe RT PCR buffer mix (400 rxn kit) 0.5 µl, NDF (12.5 µM in molecular grade water) 1 µl, NDR (12.5 µM in molecular grade water) 1 µl, LproMGB (5 µM in TE buffer) 1 µl, LproMGB 2 (5 µM in TE buffer) 1 µl, Qiagen QuantiFast RT PCR enzyme mix 0.25 µl. The cycling conditions for this test were as follows: Stage 1 - 50°C for 10 min, Stage 2 - 95°C for 5 min, Stage 3 (Step 1) - 95°C for 10 s, Stage 3 (Step 2) - 50°C for 30 s, and Stage 4 - 72°C for 30 s where steps 1 and 2 in Stage 3 were run for 40 cycles. Fluorescence was read at the end of the 2^nd^ step of Stage 3, annealing step.

### Developing standard curves

For both of the 10-fold and 2-fold dilutions of the virus samples, two different regression curves were developed using Syntax “Twoway lfit yvar xvar” with statistical package STATA 11 to make visualizing a standard curve. C_T_ values obtained from the respective dilutions were plotted on the Y-axis against their corresponding log to the dilutions at the X-axis in the stated process to produce a standard curve for each dilution.

## Results

### Overview of the vaccines

Three batches from each of five vaccine samples have been used in this experiment. The vaccines were as follows: Sample 1: Freeze-dried culture of lentogenic F strain, Sample 2: Live freeze-dried culture of LaSota strain, Sample 3: Live freeze-dried culture of lentogenic F strain, Sample 4: Live freeze-dried culture of Clone 30 strain, and Sample 5: Live freeze-dried cultures of B1 type LaSota strain.

### HA titer and thermostability

The persistence of HA titer was adopted as a measure of antigen stability. Initial HA titer was as high as up to 2^11^ which gradually declined to zero with variable time (in days) (Table-2).

**Table-2:** HA titer summary of the vaccines.

Batch	Sample 1			Sample 2			Sample 3			Sample 4			Sample 5		
					
Time	B1	B2	B3	B1	B2	B3	B1	B2	B3	B1	B2	B3	B1	B2	B3
Day 1	2^7^	2^6^	2^6^	2^9^	2^11^	2^11^	2^8^	2^6^	2^7^	2^9^	2^8^	2^9^	2^10^	2^9^	2^10^
Day 2	2^6^	2^5^	2^6^	2^9^	2^10^	2^11^	2^8^	2^6^	2^7^	2^8^	2^8^	2^9^	2^9^	2^9^	2^9^
Day 3	2^6^	2^5^	2^6^	2^9^	2^10^	2_10_	2^8^	2^6^	2^7^	2^8^	2^7^	2^9^	2^9^	2^9^	2^9^
Day 7	2^5^	2^3^	2^5^	2^8^	2^9^	2^8^	2^7^	2^5^	2^6^	2^7^	2^7^	2^8^	2^8^	2^8^	2^7^
Day 14	2^5^	2^2^	2^3^	2^6^	2^6^	2^5^	2^7^	2^3^	2^4^	2^5^	2^4^	2^5^	2^6^	2^6^	2^6^
Day 21	2^3^	0	0	2^5^	2^5^	2^5^	2^6^	2^2^	2^3^	2^4^	2^2^	2^4^	2^5^	2^4^	2^3^
Day 28	0	0	0	2^3^	2^2^	2^3^	2^3^	0	2^2^	2^2^	0	0	2^4^	2^2^	0
Day 35	0	0	0	2^2^	0	2^2^	0	0	0	0	0	0	2^1^	0	0

HA=Hemagglutination

It is clear from Table-2 that Sample 2 has the highest initial HA titer of 2^11^ followed by Sample 5 which showed an HA titer of maximum 2^10^ at the beginning. Sample 4 also showed a very good initial HA titer of 2^9^, while Samples 1 and 3 revealed an average titer of 2^6^ and 2^7^, respectively.

**Figure-1:**
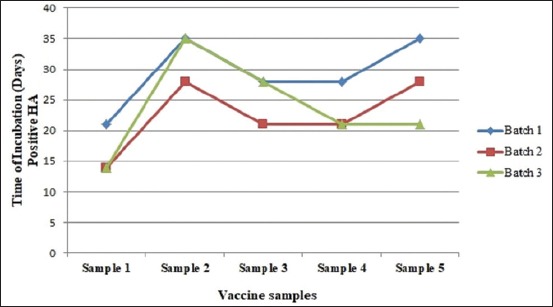
Thermostability of the vaccine samples. Sample 1: F strain, Sample 2: LaSota strain, Sample 3: F strain, Sample 4: Clone 30, Sample 5: B1 type LaSota.

Deterioration in HA ability of the vaccine samples is unfurled in Table-2. As Sample 2 has the most HA titer, this sample also persists in showing HA activity for the longer period compared to other under 37°C incubation. Comparative thermostability of three batches from five different samples is shown in Figure-1. Vaccine from Sample 2 survived more days in incubation at the experimental temperature (37°C). Two of three batches survived up to 5 weeks and another one up to 4 weeks. Samples 3, 4, and 5 showed an almost similar fashion of thermostability. Maximum survival period of these samples was 4 weeks. On the contrary, Sample 1 found to be least survived vaccine. Though, one batch among three in Sample 1 survived till 3^rd^ week.

### Validation of rRT-PCR standard curves

**Figure-2:**
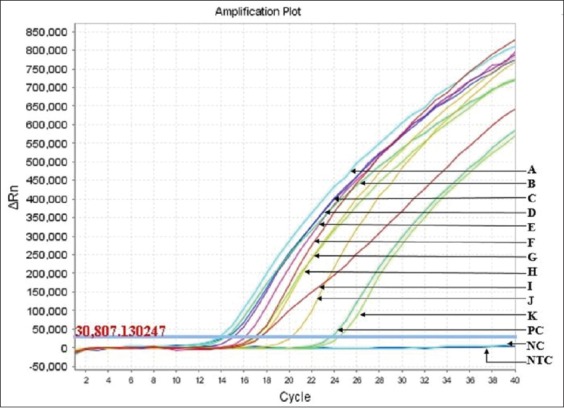
Amplification curves of the polymerase (L) gene-positive vaccine virus at 2-fold and 10-fold dilution. Two curves for each of 2-fold dilution and one curve for each of 10-fold dilution. A - 2^-1a^, B - 2^-1b^, C - 2^-2a^, D - ^−2b^, E - 2^−3a^, F - 2^−3b^, G - 2^−4a^, H - 2^−4b^, I - 10^−1^, J - 10^−2^, K - 10^−3^, PC - Positive control, NC - Negative extraction control, NTC – Non-amplification control.

**Figure-3:**
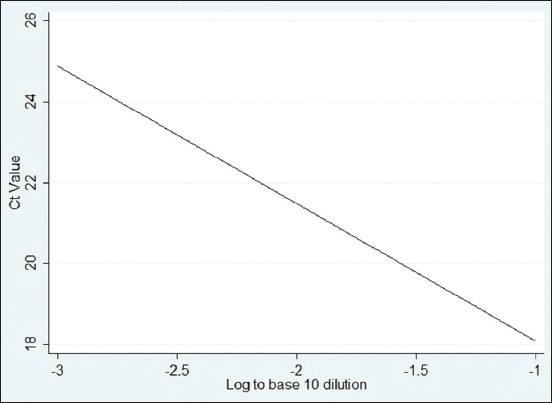
Standard curve with cycle threshold values against log (to the base 10) dilution of a Newcastle disease (ND) live vaccine containing the LaSota strain of ND virus.

**Figure-4:**
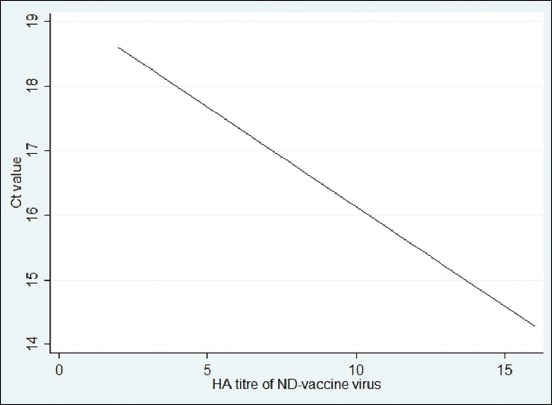
Standard curve with cycle threshold values against hemagglutination titer of a Newcastle disease (ND) live vaccine (log 2 base dilution) containing the LaSota strain of ND virus.

Two standard curves were developed on the basis of amplification curve analysis data found in rRT-PCR (Figure-2). The 1^st^ curve was prepared by plotting C_T_ values against the log 10 dilution of the vaccine, as displayed in Figure-3. Any C_T_ value from an unknown sample can be referred to the curve to find the level of virus diluted from the original virus titer in the vaccine. For example, if the C_T_ value of an unknown sample is 19, it would have been diluted at about log (to the base 10) 1.3 of its original titer as measured by any scale. The secondstandard curve developed was with the C_T_ values against the HA titer (Figure-4). This graph can be used to calculate the HA titer of a vaccine dilution by doing rRT-PCR. For example, if the C_T_ value of a vaccine dilution is 16.5, the HA titer of the virus in this dilution would be around 9. The regression equations for the first and second graphs were y=−3.535x+14.365 and y=−1.081x+13.703, respectively, meaning that, for every 3.53 cycles, the PCR product would have increased slightly above 10 times (because log [to the base 10] 3.34 equals 10) for the first curve and almost double for each cycle (because 2^1^=2) for the 2^nd^ curve. The PCR results thus indicate that the standard curves were fitted nearly (but not exactly) based on 100% PCR efficiency.

## Discussion

The study was conveyed to test the thermostability of the vaccine virus under incubation temperature (37°C) and to find a relationship between HA titer and rRT-PCR. The test was also aimed to reveal how rRT-PCR can be useful in assessing the titer is replacing the time-consuming HA testing. Five commercial live vaccines were used in this experiment, and after HA test, the vaccine that was found with higher HA titer was chosen for further RT-PCR validation.

The persistence of HA titer was adopted as a measure of antigen stability. The result of the study depicted that initial HA titers of the vaccines were variable. Sample 2 had the highest titer of 2^11^ among these. This vaccine was made from LaSota strain. Another vaccine that was made from B1 type of LaSota strain, Sample 5, showed an HA titer of 2^9^-2^10^ at the beginning. Sample 1 and 4, made of F strain, had almost the same HA titer around 2^6^ and 2^7^, respectively. The other vaccine sample made from Clone 30 strain had an initial HA titer level of 2^9^. The result showed that Sample 2 or LaSota strain vaccine has better HA titer than other live vaccine virus strain. Previous studies showed that 2- to 8-fold difference of HA titer was observed among live commercial Newcastle vaccine virus in Pakistan [25]. However, pre-temperature exposure titer log of LaSota and B1 strain was 26 and 25, respectively, in another experiment [21]. In Japan, different lentogenic strains of NDV were tested for HA titer activity and found the titer between 2^8^ and 2^11^ [26].

At an incubation temperature of 37°C, vaccine virus samples showed different lines of stability. Sample 2, LaSota strain, which had higher initial HA titer, predictably showed longer stability. A two-third batch of this sample was viable up to 35^th^ day. Only one batch from B1 type LaSota vaccine, Sample 5, showed HA activity at 35^th^ day. Other vaccine samples were found to be showing HA titer up to 21 days by Sample 1 and 28 days by Samples 3 and 4. LaSota strain virus containing live vaccine revealed to be more thermostable under the above-mentioned temperature. However, literature in this aspect was scanty in relation to the thermal exposure of virus at 37°C at incubation. The vaccine that was tested at 37°C for the thermostability of the virus isolates as per Aini [27] revealed that HA titer of that vaccine virus was 2^9^ for V4 strain after 29 days of incubation.

The variability in the titers of the vaccine virus strains at the beginning of the study could have an effect on seeing uneven period of thermostability among them. While acknowledging this limitation, it was also an aim of the study to see the titers of NDV in the most common ND vaccines being commercially marketed in Bangladesh.

Two types of vaccine are considered thermostable: Australian V4 and I2, both are produced from live, avirulent strain [9,14]. Australia V4 had both thermostability and immunogenicity and was specifically developed to be used in village chicken. It is administered in coated pelleted feed [28].

A thermostable vaccine enables distributors and users to reduce the problems associated with inadequate cold chains in the field. Instead of that, a thermostable vaccine must still be treated with some of the respects as it is a biological product, that the vaccine being exposed to sunlight, and frequent shifts in temperature and still expect it to remain active [29]. Any break in this chain removes any guarantee of effective protection against the disease [22,30].

To check the validity of rRT-PCR in assessing the HA titer, C_T_ value achieved from the PCR and HA titer along with the dilution of the vaccine virus sample was analyzed in different ways. It is obvious that C_T_ value is inversely proportionate to the dilution of the virus. This experiment also found the same pattern. As C_T_ value increased, HA titer decreased gradually. However, validation of rRT-PCR of a virus for rapid quantification of HA titer in vaccine is not well established yet. Therefore, in this study, we tried to develop and validate an rRT-PCR method for rapid quantification of NDV in vaccine sample. The PCR method was operated based on targeting polymerase (L) gene of APMV-1 as the sensitivity of this method is much higher than other gene targeting PCR method [24].

Comparing C_T_ value against the HA titer of both 2-fold and 10-fold diluted vaccine samples, the correlation coefficients were 0.9753 and 0.9638, respectively. Hence, the C_T_ value can be 97.53% accurate to determine the virus titer during 2-fold dilution, while it is 96.38% in 10-fold.

In the case of dilution of the vaccine virus, C_T_ value can be predicted up to 99.35% accuracy from the dilution when dilution factor is 10-fold. If the dilution factor is 2-fold, then increase or decrease of the threshold or C_T_ value of the virus sample can be assumed with appropriateness of 97.76%. A C_T_ value as obtained from rRT-PCR could be the reflection of the titer including live viruses plus intact RNA from inactivated virus if remained present at all. It was difficult to differentiate them by rRT-PCR. However, plotting C_T_ values against HA titers to the second standard curve developed could be the way to know the tentative live virus titer subtracted from the amount of intact RNA of NDV.

The most important observation was done by finding the difference of C_T_ value if the HA titer of the vaccine virus sample changed. When HA titer falls, it clearly indicates a decrease in the amount of virus in the vaccine. In this experiment, we found that HA titer was 97.41% precise against the change of the C_T_ value difference in case of 2-fold dilution, while it is 96.38% in case of 10-fold. Hence, the change of the C_T_ value of the sample can determine the virus concentration and thus assure the validity of the vaccine. As an rRT-PCR result is based on the number of RNA of the virus present in a matrix, the result of the study would be similar to any of the lentogenic virus strains of NDV being used as a commercial ND vaccine. Results of any further studies with a mesogenic or velogenic strain would be interesting to know to verify whether such results are reproducible to the result obtained in the present study.

This rRT-PCR assay based on the L-gene has the potential to decrease the time taken to confirm the concentration of suspected virus sample, thus determining the vaccine potential with a high degree of certainty [24]. Although serological identification followed by *in vivo* pathogenicity testing still remains as the gold standard, RT-PCR offers a reliable tool for quick detection of NDV. This method not only takes a shorter time to produce a result but also has more accuracy, precision, specificity, and robustness [31]. Specifically, the method was suitable for the quantification of virus titer in the vaccine sample, and it is a rapid method.

## Conclusion

Among five live vaccines of different strains, one that prepared from LaSota strain showed the maximum days of positive HA (up to 35 days) under incubation at 37°C. This vaccine also showed higher initial HA titer of 2^11^. Vaccine made from F strain survived least among these all and also showed the lowest HA titer. It indicates that vaccines from different strains have variable adaptability to the temperature. Other factors may be involved in determining the titer of any live virus vaccine. However, this study would help to understand the pattern of decline in the HA titer of the vaccines. C_T_ value of the vaccine virus sample, determined by rRT-PCR, suggests that this value varies with the dilution of virus. The two rRT-PCR standard curves developed can be used to guess the level of virus titer at any dilution from its titer at the originally undiluted stage and to estimate the level of HA titer in any diluted live ND vaccine. The curves were based on nearly (but not exactly) 100% PCR efficiency. Any future study using mesogenic or velogenic strains of this virus will help better understanding the relationship between rRT-PCR and HA titer. It will aid the vaccine manufacturers as well as researchers in deciding HA titer in the shortest possible time with much more accuracy and precision.

## Authors’ Contributions

PKD and PKB: Conception and design of the study. PKD, ADu, ADa, and MSJ: Conducted the experiment, analyzed, and interpreted the data. PKD, HB, and PKB: Drafting and revising the manuscript critically for important intellectual content. All authors have read and approved the final manuscript.
